# Effect of Perioperative Lipid Status on Clinical Outcomes after Cardiac Surgery

**DOI:** 10.3390/cells10102717

**Published:** 2021-10-11

**Authors:** Maks Mihalj, Paul Philipp Heinisch, Markus Huber, Joerg C. Schefold, Alexander Hartmann, Michael Walter, Elisabeth Steinhagen-Thiessen, Juerg Schmidli, Frank Stüber, Lorenz Räber, Markus M. Luedi

**Affiliations:** 1 Department of Cardiovascular Surgery, Inselspital, Bern University Hospital, 3010 Bern, Switzerland; maks.mihalj@insel.ch (M.M.); paulphilipp.heinisch@extern.insel.ch (P.P.H.); juerg.schmidli@insel.ch (J.S.); 2 Department of Intensive Care Medicine, Inselspital, Bern University Hospital, University of Bern, 3010 Bern, Switzerland; joerg.schefold@insel.ch; 3 Department of Anaesthesiology and Pain Medicine, Inselspital, Bern University Hospital, University of Bern, 3010 Bern, Switzerland; markus.huber@insel.ch (M.H.); frank.stueber@insel.ch (F.S.); 4 Department of Congenital and Pediatric Heart Surgery, German Heart Center Munich, Technische Universität München, 80636 Munich, Germany; 5 Institut für Klinische Chemie und Laboratoriumsmedizin, Universitätsmedizin Rostock, 18057 Rostock, Germany; alexander.hartmann2@med.uni-rostock.de (A.H.); michael.walter@med.uni-rostock.de (M.W.); 6 Department of Endocrinology and Metabolic Medicine, Division of Lipid Metabolism, Charité–Universitätsmedizin Berlin, 13353 Berlin, Germany; elisabeth.steinhagen-thiessen@med.uni-rostock.de; 7 Department of Cardiology, Inselspital, Bern University Hospital, 3010 Bern, Switzerland; lorenz.raeber@insel.ch

**Keywords:** lipid, LDL, HDL, triglycerides, cholesterol, cardiac surgery, cardiopulmonary bypass

## Abstract

Patients undergoing cardiac surgery are at increased cardiovascular risk, which includes altered lipid status. However, data on the effect of cardiac surgery and cardiopulmonary bypass (CPB) on plasma levels of key lipids are scarce. We investigated potential effects of CPB on plasma lipid levels and associations with early postoperative clinical outcomes. This is a prospective bio-bank study of patients undergoing elective cardiac surgery at our center January to December 2019. The follow-up period was 1 year after surgery. Blood sampling was performed before induction of general anesthesia, upon weaning from cardiopulmonary bypass (CPB), and on the first day after surgery. Clinical end points included the incidence of postoperative stroke, myocardial infarction, and death of any cause at 30 days after surgery as well as 1-year all-cause mortality. A total of 192 cardiac surgery patients (75% male, median age 67.0 years (interquartile range 60.0–73.0), median BMI 26.1 kg/m^2^ (23.7–30.4)) were included. A significant intraoperative decrease in plasma levels compared with preoperative levels (all *p* < 0.0001) was observed for total cholesterol (TC) (Cliff’s delta *d*: 0.75 (0.68–0.82; 95% CI)), LDL-Cholesterol (LDL-C) (*d*: 0.66 (0.57–0.73)) and HDL-Cholesterol (HDL-C) (*d*: 0.72 (0.64–0.79)). At 24h after surgery, the plasma levels of LDL-C (*d*: 0.73 (0.650.79)) and TC (*d*: 0.77 (0.69–0.82)) continued to decrease compared to preoperative levels, while the plasma levels of HDL-C (*d*: 0.46 (0.36–0.55)) and TG (*d*: 0.40 (0.29–0.50)) rebounded, but all remained below the preoperative levels (*p* < 0.001). Mortality at 30 days was 1.0% (*N* = 2/192), and 1-year mortality was 3.8% (*N* = 7/186). Postoperative myocardial infarction occurred in 3.1% of patients (*N* = 6/192) and postoperative stroke in 5.8% (*N* = 11/190). Adjusting for age, sex, BMI, and statin therapy, we noted a protective effect of postoperative occurrence of stroke for pre-to-post-operative changes in TC (adjusted odds ratio (OR) 0.29 (0.07–0.90), *p* = 0.047), in LDL-C (aOR 0.19 (0.03–0.88), *p* = 0.045), and in HDL-C (aOR 0.01 (0.00–0.78), *p* = 0.039). No associations were observed between lipid levels and 1-year mortality. In conclusion, cardiac surgery induces a significant sudden drop in levels of key plasma lipids. This effect was pronounced during the operation, and levels remained significantly lowered at 24 h after surgery. The intraoperative drops in LDL-C, TC, and HDL-C were associated with a protective effect against occurrence of postoperative stroke in adjusted models. We demonstrate that the changes in key plasma lipid levels during surgery are strongly correlated, which makes attributing the impact of each lipid to the clinical end points, such as postoperative stroke, a challenging task. Large-scale analyses should investigate additional clinical outcome measures.

## 1. Introduction

Dyslipidemia is a known risk factor for cardiovascular events, including stroke, myocardial infarction (MI), kidney disease, and calcifying vascular disease, to name only a few. Elevated levels of total cholesterol (TC), low-density lipoprotein (LDL), cholesterol, triglycerides (TG), and low levels of high-density lipoprotein (HDL) cholesterol are commonly associated with an elevated risk of atherosclerotic plaque formation and thus with development and progression of cardiovascular disease [1,2,3,4]. Medical therapy aimed at lowering plasma levels of TC, LDL-Cholesterol (LDL-C), and TG represents one of the main therapeutic strategies used for patients with cardiovascular disease [1,4,5,6,7]. The value of statin use in patients undergoing cardiac surgery is disputed, and some studies suggest statin is associated with increased occurrence of postoperative acute kidney failure and hemorrhagic stroke [4,5,8,9]. Specifically in cardiac surgery patients, dyslipidemia is known to influence graft patency after coronary artery bypass grafting surgery (CABG) [10,11,12] or after percutaneous coronary intervention (PCI), [1,3,4] and has been associated with calcifying aortic stenosis [4]. Patients undergoing cardiac surgery often have an extensive portfolio of risks for cardiovascular diseases, including altered lipid status, for which they often receive lipid-lowering therapy [8]. Lipoprotein (a) levels are significantly reduced during cardiopulmonary bypass (CPB), but little is known about the effects of cardiac surgery and cardiopulmonary bypass (CPB) on the plasma level alterations in other key lipids. In this prospective biobank study, we investigated the effect of cardiac surgery on key plasma lipid levels and how these are associated with early postoperative clinical outcomes and complications.

## 2. Materials and Methods

### 2.1. Cohort Description

We performed an observational cohort study with a prospective sample of 192 adult patients from the Bern Perioperative Biobank (ClinicalTrials.gov NCT04767685) who underwent cardiac surgery at the Bern University Hospital between January 2019 and December 2019. Patients were included if they were scheduled for elective cardiac surgery and provided written informed consent. Cardiac surgical procedures included CABG; replacement or repair of aortic (AVR), mitral (MVR), and tricuspid valves (TVR); as well as surgery of the ascending aorta or aortic arch. All patients received median sternotomy and cardiopulmonary bypass, either with conventional extracorporeal circulation circuits (CECC) or minimally invasive extracorporeal circulation circuits (MIECC). Patients undergoing emergent surgeries and women with suspected or confirmed pregnancy were excluded.

### 2.2. Collection and Analysis of Blood Samples

Blood samples (whole blood and plasma) were collected at 24 h preoperatively (baseline), 30 min after induction of anesthesia (pre-operative), upon weaning from CPB (intra-operative), and 24 h after surgery (post-operative) and were stored at the Bern Liquid Biobank. Biochemical markers were analyzed at the institutional laboratory of the Institute of Clinical Chemistry and Laboratory Medicine, University Medical Center Rostock, Rostock, Germany. An automated DxC 700 AU chemistry analyzer (Beckman-Coulter, Brea, CA, USA), wielding Beckman–Coulter reagents, was used to measure the concentrations of relevant indicators, which included LDL-C (REF: OSR6187), TC (REF: OSR6116), TG (REF: OSR60118), and HDL-Cholesterol (HDL-C) (REF: OSR6187). Clinical data were collected from internal hospital records (Dendrite Clinical Systems Ltd., Henley on Thames, UK) and updated for inclusion in the standardized database using the Research Electronic Data Capture (REDCap) system. Patients were followed up until postoperative day 30, and all-cause mortality was evaluated at one year after surgery. We investigated the change in plasma lipid levels (TC, LDL-C, HDL-C, and TG) before, during, and after cardiac surgery, including how this change was associated with clinical endpoints of postoperative stroke, myocardial infarction, and death of any cause at 30 days after surgery, as well as overall survival one year after surgery. As a subgroup analysis, we compared patients with ongoing statin therapy to those not receiving statin therapy. Local ethics committee approval was obtained for sample collection (KEK Nr. 2018-01272) and data analysis (KEK Nr. 2019-2000). Written informed consent was obtained from all patients. The study was performed in adherence to the Declaration of Helsinki.

### 2.3. Statistical Analysis

Continuous variables were examined with the Shapiro–Wilks test and are presented as mean with standard deviation (SD) in case of normally distributed variables and median with interquartile range (IQR) otherwise. Categorical variables are presented with counts and percentages. Group comparisons were based on a regular chi-square test for categorical variables and on a permutation chi-square test (with 2000 samples) when the expected counts in some cells were lower than 5. For continuous variables, group comparisons were based on Student’s *t*-test for normally distributed variables and on the Wilcoxon rank-sum test otherwise.

Lipid values at different time points are presented with medians and IQR. Pairwise post-hoc comparisons of lipid levels are illustrated using Cliff’s delta (*d*) as effect size and with the Wilcoxon signed-rank test with a Bonferroni adjustment for multiple comparisons as a test of significance. The strength of the pairwise correlations for the change in lipid levels from preoperative to intraoperative and from preoperative to postoperative levels is illustrated as scatterplots alongside the model fit of a linear regression. Pearson correlation coefficients and *associated p*-values are shown for each pairwise correlation. Bivariate associations of the change in lipid levels from pre-operative to post-operative values for each surgical characteristic (i.e., CPB time) are illustrated using box plots (in case of categorical variables) and with locally estimated scatterplot smoothing (LOESS) for continuous variables.

The binary clinical end points were incidence of postoperative stroke, myocardial infarction, death of any cause at 30 days after surgery, as well as overall survival one year after surgery. These are presented with counts and percentages as well as the inferred 95% Clopper–Pearson intervals. The association of preoperative lipid values and their change during the operation (postoperative minus preoperative) with the two end points, postoperative stroke and survival after one year, are computed with (i) univariable logistic regression; (ii) univariable logistic regression adjusting for confounders age, sex, BMI, and statin therapy; (iii) multivariable elastic net regression of all lipid variables; and (iv) multivariable elastic net regression of all lipid variables adjusted including for CPB time as a potential confounder (note that there are no formal 95% confidence intervals associated with the regression coefficients of an elastic net model; thus, only the estimates of the odds ratios are shown). The elastic net approach [13] performs a penalized regression with the ability to handle correlated predictors in a more balanced way than traditional regression approaches such as logistic regression, and its optimal parameter values are found by minimizing the cross-validation error. A *p*-value < 0.05 was considered statistically significant. All computations were performed using the R software environment (R version 4.0.2; R Core Team (2020)).

## 3. Results

A total of 192 patients were included in the study. Patient demographics are presented in Table 1. Most patients were male (75.5%), and median age was 67.0 years (interquartile range 60.0–73.0), with a median BMI of 26.1 kg/m2 (23.7–30.4). Most patients had a low risk of perioperative mortality, with median EuroSCORE II scores of 1.73% (0.90–2.93). The most common cardiovascular risk factors included arterial hypertension (68.4%), dyslipidemia (58.1%), smoking (47.4%), obesity (27.1%), chronic kidney disease (22.4%), and diabetes mellitus (18.2%). A history of previous MI was reported in 10.5%, and significant carotid disease was present in 7.3%. The most commonly used preoperative medications were statins (54.7%), followed by acetylsalicylic acid (ASA; 47.9%), beta blockers (44.8%), angiotensin-converting enzyme inhibitors (ACEI; 41.1%), and angiotensin receptor blockers (ARB; 24.5%. Median left-ventricular ejection fraction (EF) was 60.0% (55.0–65.0). Preoperative median lipid plasma levels were above the target recommended by the current European Society of Cardiology (ESC) guidelines [4] (Table 1 and Table 2). The most common procedures included AVR (44.8%) and CABG (40.1%), followed by MVR (23.4%) and ascending aortic replacement (19.8%), with a median CPB time of 104 min (80.0–132). A minimally invasive extracorporeal circulation (MiECC circuit) was used in isolated CABG cases only (22.4%).

### 3.1. Subgroup Analysis of Statin Therapy

For the subgroup analysis, patients were grouped into those receiving lipid-lowering statin therapy (*N* = 105; 54.7%) and those without statin therapy (*N* = 87; 45.3%), respectively. Although groups were comparable in size, fewer patients on statin therapy were female (15.2% vs. 35.6%, *p* = 0.002) and had a higher comorbidity profile, including higher rates of diabetes mellitus (26.7% vs. 8.05%, *p* = 0.002), arterial hypertension (79.8% vs. 54.7%, *p* < 0.001), dyslipidemia (79.8% vs. 32.2%, *p* < 0.001), and prior myocardial infarction (17.1% vs. 2.3%, *p* = 0.04). Significantly more patients under statin therapy had chest pain equivalent to Canadian Cardiovascular Society angina score ≥ 2 (28.6% vs. 8.0%, *p* < 0.001), and accordingly, they underwent CABG or AVR surgery more often (63.6% vs. 36.4%, *p* < 0.001). However, patients without statin therapy had a higher risk of perioperative mortality (logistic EUROSCORE 5.13% (3.62–8.81) vs. 3.86% (1.78–7.62), *p* = 0.01) and more often received combined valve or aortic procedures (59.1% vs. 40.9%, *p* < 0.001), using CECC in 97.7% of cases (compared to 61.0% in the statin group, *p* < 0.001). Patients under statin therapy had significantly lower levels of TC (3.92 mmol/L ± 0.94 vs. 5.03 mmol/L ± 1.04, *p* < 0.001), LDL-C (2.34 mmol/L (1.98–2.70) vs. 3.25 mmol/L (2.74–3.85), *p* < 0.001), and HDL-C (1.08 mmol/L (0.88–1.31) vs. 1.20 mmol/L (1.00–1.42), *p* = 0.012). No significance in TG was observed between groups (*p* = 0.323). Further patient characteristics, procedural data, and outcomes are shown in Supplementary Table S1.

### 3.2. Perioperative Alterations in Lipid Levels

**Figure 1:**
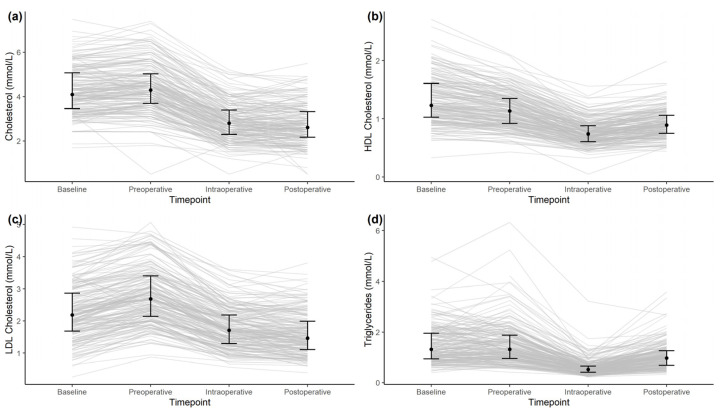
Time series of cholesterol (**a**), HDL-C (**b**), LDL-C (**c**), and triglycerides (**d**) for the baseline, pre-operative, intra-operative, and post-operative time points. Shown are medians and interquartile ranges at the different time points (black dots and black lines) and time series of individual patients (grey lines).

During cardiac surgery, a significant intraoperative decrease in plasma levels from preoperative levels (all *p* < 0.0001) was observed for TC (Cliff’s delta *d*: 0.75 (0.68–0.82; 95% CI)), LDL-C (*d*: 0.66 (0.57–0.73)), and HDL-C (*d*: 0.72 (0.64–0.79)). At 24 h after surgery, the plasma levels of LDL-C (*d*: 0.73 (0.65–0.79)) and TC (*d*: 0.77 (0.69–0.82)) continued to decrease compared to preoperative levels, while the plasma levels of HDL-C (*d*: 0.46 (0.36–0.55)) and TG (*d*: 0.40 (0.29–0.50)) rebounded, but all remained below the preoperative levels (*p* < 0.001). When analyzing the difference between preoperative and postoperative plasma lipid levels, a significant decrease in all measured lipids was observed (*p* < 0.001). Further results are presented in Table 2 and Figure 1.

**Figure 2:**
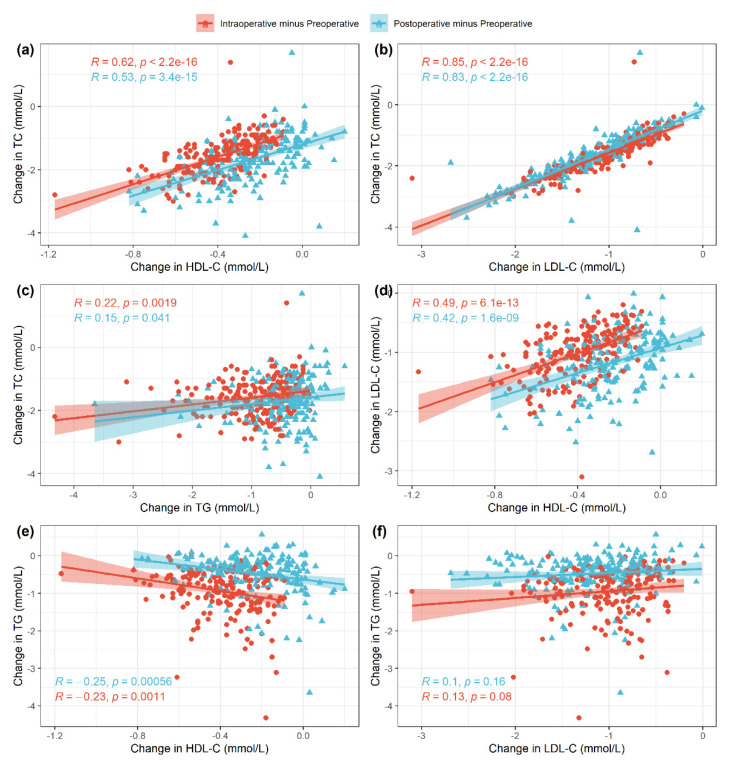
Pairwise associations across the different variables with associated Pearson correlation coefficients and *p*-values: TC and HDL-C (**a**), TC and LDL-C (**b**), TC and TG (**c**), LDL-C and HDL-C (**d**), TG and HDL-C (**e**), TG and LDL-C (**f**). For each patient, the changes from preoperative to intraoperative values (red) and from preoperative to postoperative values (blue) are shown.

When analyzing for correlations between individual lipids, we calculated the difference in plasma levels between preoperative and intraoperative values as well as between preoperative and postoperative values (Figure 2), respectively. The strongest positive linear correlation was observed between changes in LDL-C and TC (Pearson correlation coefficient *r* = 0.85 and *r* = 0.83, *p* < 0.001; Figure 2b), followed by strong positive linear correlation between TC and HDL-C (*r* = 0.62 and *r* = 0.53, *p* < 0.001; Figure 2a) and medium positive linear correlation between changes in LDL-C and HDL-C (*r* = 0.49 and *r* = 0.42, *p* < 0.001; Figure 2d). A weak negative linear correlation was observed between changes in TG and HDL-C (*r* = −0.25 and *r* = −0.23, *p* < 0.05; Figure 2e) as well as between changes in TG and LDL-C (*r* = 0.1 and *r* = 0.13, *p* > 0.05; Figure 2f). The latter correlation was not significant, however.

**Figure 3:**
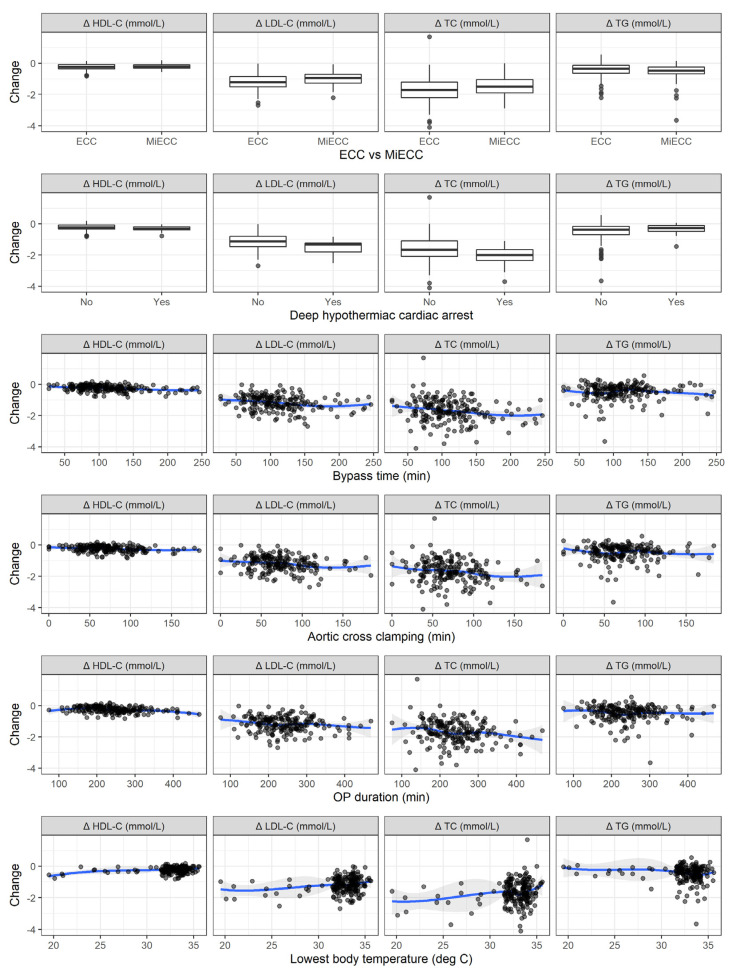
Associations between the change in lipid levels and surgical characteristics, stratified according to statin therapy.

The change in lipid levels was further investigated for individual surgical characteristics, represented in Figure 3. In terms of linear associations, a longer bypass time was associated with a significant decrease in TC (*p* = 0.007), HDL-C (*p* < 0.001), and LDL-C (*p* = 0.003). Longer aortic cross clamping was found to be associated with a significant decrease in TC (*p* = 0.015), HDL-C (*p* = 0.002), and LDL-C (*p* = 0.004). Longer operations were associated with larger decreases only in TC (*p* = 0.012) and HDL-C (*p* < 0.001). Higher lowest body temperatures were associated with smaller drops in TC (*p* = 0.004), HDL-C (*p* = 0.004), and LDL-C (*p* = 0.001).

### 3.3. Clinical Outcomes

**Figure 4:**
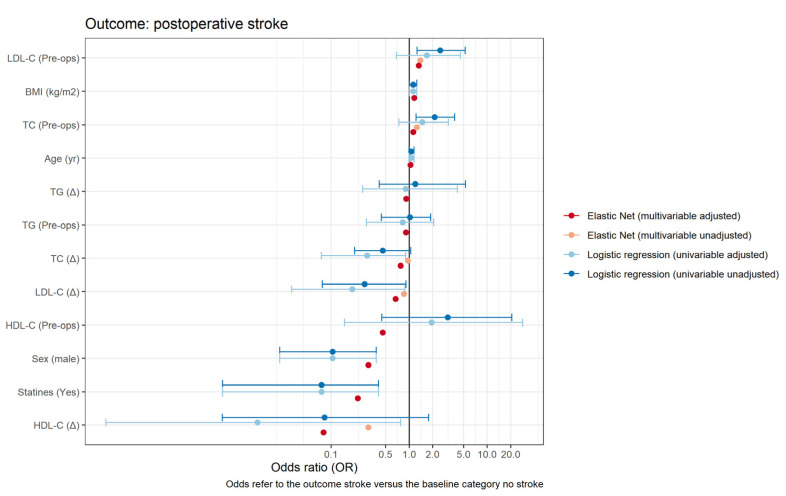
Forest plot showing the estimates of the odds ratio of various predictors on the outcome postoperative stroke. For the logistic regression models, 95% confidence intervals are shown as well (adjusted values). No formal confidence intervals are computed for the elastic net regressions.

Overall, two patients died within 30 days after surgery (1.04%, *N* = 192), and seven patients within one year after surgery (3.8%, *N* = 186). Postoperative myocardial infarction occurred in six patients (3.12%, *N* = 186), and postoperative stroke was observed in 11 patients (5.7%, *N* = 186). Clinical outcomes are summarized in Table 3. We found significantly lower risk of postoperative stroke in patients who received statin therapy (crude OR 0.08 (95% CI 0.00, 0.41), *p* = 0.015; Table 4, Figure 4). Note, however, that this constitutes a crude estimate without accounting for possible imbalances in other confounders with respect to the administration of a statin therapy. This risk reduction continued to be observed in an adjusted multivariable elastic net regression model (adjusted OR 0.15; Table 5). No difference in preoperative demographics was observed for other clinical endpoints (Table S2).

### 3.4. Association between Lipids and Postoperative Stroke and Overall Survival

**Figure 5:**
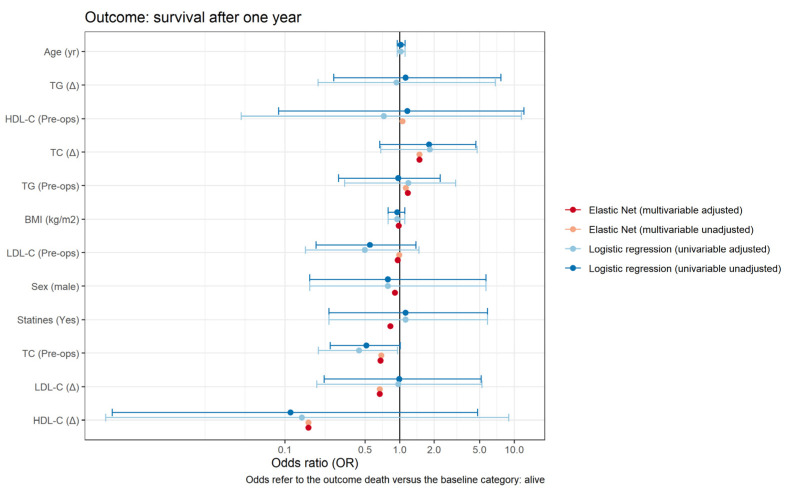
Forest plot showing the estimates of the odds ratio of various predictors on the outcome overall survival after one-year follow-up. For the logistic regression models, 95% confidence intervals are shown as well (adjusted values). No formal confidence intervals are computed for the elastic net regressions.

The association between lipids and postoperative stroke is presented in Table 4. A significantly elevated risk of developing postoperative stroke was observed by preoperative levels of TC and LDL-C (crude OR 2.12 (1.23–3.82), *p* = 0.008; and OR 2.51 (1.27– 5.21), *p* = 0.01, respectively). However, after adjusting for the confounder’s age, gender, BMI, and statin therapy, we found no evidence of significance. In the adjusted model, a protective effect was observed for pre-to-post-operative changes in TC (aOR 0.29 (0.07–0.90), *p* = 0.047), in LDL-C (aOR 0.19 (0.03–0.88), *p* = 0.045), and in HDL-C (aOR 0.01 (0.00–0.78), *p* = 0.039). This protective effect continued to be observed in the adjusted multivariable elastic net regression model, although it was less evident (Table 4). We observed no significant increase or decrease in the risk of dying within one year after cardiac surgery between the pre-operative plasma lipid levels or their respective perioperative changes (Table 5, Figure 5). Adjusting for CPB time as a potential confounder did not reveal a significant impact (Supplemental Table S3). Missing data are presented in Supplemental Table S4, descriptive statistics of cholesterol, HDL-C, LDL-C, and triglycerides at the different time points for those patients only who did not receive any blood transfusion perioperatively in Supplemental Figure S5 and Supplemental Table S6 and the association between individual lipids (in units [mmol/L]) and the outcome postoperative stroke in Supplemental Table S7.

## 4. Discussion

We observed a significant decline in levels of key plasma lipid mediators during cardiac surgery, with the most significant decrease being observed in LDL-C and TC and the weakest effect observed in HDL-C. The overall drop in LDL-C, TC, and HDL-C was associated with the strongest risk reduction for postoperative stroke as well as with statin therapy. All plasma lipid levels remained significantly lowered at 24 h after surgery. The lipids with the strongest perioperative linear correlation were LDL-C and TC, TC and HDL-C, as well as LDL-C and HDL-C. A perioperative decrease in plasma lipid levels was not associated with a significant reduction or increase in the risk of death at one year after cardiac surgery.

Little is known about the dynamics of lipid plasma levels during cardiac surgery and how this affects clinical outcomes, although a drop in plasma levels of LDL-C and TC has previously been observed up to five days after cardiac surgery [5,14,15,16]. Our results confirm that a highly significant decrease in plasma levels of TC, LDL-C, and HDL-C occurred during and after CPB. This decrease remained significantly lowered to the pre-operative plasma levels at 24 h after surgery in all investigated lipids. While the exact mechanisms remain to be determined, the most probable cause is the extracorporeal circulation and its inherent membrane oxygenator as elemental parts of the CPB circuit used for cardiac surgeries. In the subgroup analysis, no significant difference of decrease was observed between CECC and MiECC circuits. This may indicate that a closed-loop system with a reduced reservoir (MiECC), no blood-air contact, and a centrifugal pump, such as a MiECC, is known to reduce SIRS reaction post cardiac surgery, does not significantly alternate the plasma lipid levels compared to the CECC systems. It may also indicate that the reason for the significant decrease in lipids is multifactorial (e.g., loss of free circulating lipids or increased cellular reuptake of circulating lipids during surgery). Interestingly, however, plasma levels of HDL-C were only marginally affected by the investigated intraoperative variables (CPB system, CPB time, temperature, DHCA, and overall surgical time). This might imply that an additional modifying factor is present in all cases (e.g., the membrane oxygenator), but this needs to be investigated further.

While statin therapy seemed to offer the strongest protection against postoperative stroke in the unadjusted model, we found no evidence for significance when we adjusted for the confounders age, gender, BMI, and statin therapy. In the adjusted model, however, the strongest protective effect was observed in the intraoperative drop in HDL-C, with aOR 0.01 (0.00–0.78), *p* = 0.039). This contradicts the recent findings that lower levels of HDL-C in patients after acute coronary syndrome are associated with an increased risk of cardiovascular events, including hemorrhagic stroke [17,18]. Nonetheless, this effect should be interpreted carefully, as changes in the key plasma lipid level during surgery are strongly correlated, which makes it challenging to attribute the impact of each lipid to clinical endpoints, such as postoperative stroke. Additional large-scale analyses seem required to investigate potential additional clinical outcome measures.

An important observation is that, although statin therapy had been established in roughly 55% of the patients, the median preoperative plasma levels of all lipids were above the recommended target range set in the 2019 European Society of Cardiology (ESC) guidelines [4]. Dyslipidemia—especially elevated LDL-C plasma levels—has been associated with an increased risk of reduced graft patency after CABG surgery, and rapid reductions of LDL-C plasma levels have been associated with favorable outcomes and improved overall survival after acute coronary syndrome [1,19,20] and CABG surgery [10,11,12,21,22,23]. However, low plasma level of lipids have been previously associated with increased risk of hemorrhagic stroke [18]. As 10 out of 11 patients who suffered postoperative strokes received no statin therapy, it may indicate that statin therapy has a protective effect against postoperative stroke in the setting of rapid decrease in lipid plasma levels observed during CPB. Furthermore, statin therapy and a perioperative decrease in HDL-C plasma levels was associated with the lowest risk for postoperative stroke, indicating a potentially protective effect after cardiac surgery.

However, when analyzing the end point all-cause mortality one year after surgery, a significant perioperative decrease in HDL-C and LDL-C was associated with the lowest net risk. Inversely, perioperative decreases in TC and TG were observed to have the highest net protective effect against death. Future studies are needed to investigate this correlation, and these results should be interpreted with caution. As death occurred in only seven patients (3.6%), the statistical significance is limited. Similarly, while only two patients (1.04%) experienced postoperative myocardial infarction, the low event rate is insufficient to confidently perform a risk analysis. At first glance, it is surprising that a low HDL cholesterol concentration correlates with a better perioperative outcome. Yet, it has long been known that all lipid fractions are considered negative acute phase reactants [24,25]. This implies that their concentration decreases in acute or chronic stress. It was also described that the potentially protective reverse cholesterol transport is disturbed in an acute phase response [26] and can thus explain the reduced HDL cholesterol in the here-described study. Furthermore, it is known that long-term chronic inflammation, for example, induced by chronic infections [27] or rheumatoid arthritis [28], increases the cardiovascular risk. In this respect, the results shown here are remarkable. Apparently, a short-term stress response, at least in this setting, has a cardioprotective effects despite the short-term inflammatory response and despite the disturbed reverse cholesterol transport, which may point to a different significance of short-term and chronic stress in atherogenicity and/or thrombogenicity.

This single-center study showing significant risk reduction at one year after cardiac surgery has inherent limitations. Due to strict inclusion and exclusion criteria, only roughly a fifth of cardiac surgery patients were included, adding to potential selection bias. Due to the prospective design of the study and data acquisition, however, the treatment bias is minimized. As the investigated lipids correlate strongly with one another, it is statistically challenging to differentiate between a single variable effect and a joint effect of multiple variables on the clinical outcome investigated. For this purpose, the elastic net regression model was chosen to further investigate the net effect of a single variable. While we adjusted the analysis for the potential confounders age, gender, BMI, and statin therapy, we were limited in our ability to control for other confounding more rigorously due to limited sample size and low event rates. Hence, as is generally true in observational research, our results do not support causal interferences or draw conclusions but should rather be interpreted in terms of associations. Prior sample size and power analysis calculation were not performed; thus, results should be interpreted with caution and confirmed in a larger study.

## 5. Conclusions

In conclusion, cardiac surgery induces a significant sudden drop in levels of key plasma lipids. This effect was pronounced during the operation, and levels remained significantly lowered at 24 h after surgery. The intraoperative drop in LDL-C, TC, and HDL-C was associated with a protective effect against occurrence of postoperative stroke in adjusted models, with the strongest protective effect observed in lowering of HDL-C levels. These results should be interpreted with caution, however, as the changes in the key plasma lipid level during cardiac surgery are strongly correlated, which makes it challenging to attribute the impact of each lipid to the clinic endpoints, such as postoperative stroke. Additional large-scale analyses are needed to investigate potential additional clinical outcome measures.

## Figures and Tables

**Table 1:** Baseline patient demographics and perioperative characteristics.

Demographics and Perioperative Characteristics	All Patients (*N* = 192)
**Demographics**	
**Age** (y)	67.0 (60.0; 73.0)
**Height** (cm)	173 (8.71)
**Weight** (kg)	80.4 (70.0; 90.1)
**BMI** (kg/m^2^)	26.1 (23.7; 30.4)
**Sex** (Male)	145 (75.5%)
**Comorbidities**	
**Diabetes** (Yes)	35 (18.2%)
**Diabetes on insulin**	
No	24 (68.6%)
Yes	11 (31.4%)
**Hypertension***^†^* (Yes)	130 (68.4%)
**Dyslipidemia***^†^* (Yes)	111 (58.1%)
**Nicotine** *^†^*	
Former smoker	49 (26.1%)
Non-smoker	97 (51.6%)
Smoker	42 (22.3%)
**Obesity** (Yes)	52 (27.1%)
**Preoperative renal disease** (Yes)	43 (22.4%)
**Peripheral vascular disease** *^†^*	
No	167 (93.8%)
Stage 1	4 (2.25%)
Stage 2	4 (2.25%)
Stage 3	1 (0.56%)
Stage 4	2 (1.12%)
**Carotid disease** *^†^*	
<50%	1 (0.58%)
>90%	3 (1.75%)
50–69%,	9 (5.26%)
70–89%	2 (1.17%)
no	156 (91.2%)
**Myocardial infarction** *^†^*	
No MI	171 (89.5%)
MI 0–7 days before operation	3 (1.57%)
MI 8–90 days before operation	8 (4.19%)
MI > 90 days before operation	9 (4.71%)
**COPD***^†^* (Yes)	23 (12.1%)
**NYHA** *^†^*	
1	60 (31.4%)
2	90 (47.1%)
3	38 (19.9%)
4	3 (1.57%)
**CCS** *^†^*	
0	118 (62.4%)
1	34 (18.0%)
2	25 (13.2%)
3	9 (4.76%)
4	3 (1.59%)
**Ejection fraction** *^†^*	60.0 (55.0; 65.0)
**EuroSCORE2** *^†^*	1.73 (0.90; 2.93)
**Baseline lipid plasma levels**	
**Cholesterol** (mmol/L)	4.42 (1.13)
**HDL-C** (mmol/L)	1.13 (0.92; 1.35)
**LDL-C** (mmol/L)	2.68 (2.14; 3.40)
**Triglycerides** (mmol/L)	1.33 (0.97; 1.88)
**Perioperative characteristics**	
**Aortic valve surgery** (Yes)	86 (44.8%)
**Mitral valve surgery** (Yes)	45 (23.4%)
**Tricuspid valve surgery** (Yes)	17 (8.85%)
**Coronary artery bypass surgery** (Yes)	77 (40.1%)
**Ascending aortic surgery** (Yes)	38 (19.8%)
**Aortic arch surgery** (Yes)	11 (5.73%)
**ECC or MiECC**	
ECC	149 (77.6%)
MiECC	43 (22.4%)
**Bypass time** (min)	104 (80.0; 132)
**Aortic cross clamping** (min)	68.5 (52.0; 91.8)
**Lowest body temperature** (deg C)	33.2 (32.1; 33.8)
**Deep hypothermic cardiac arrest***^†^* (Yes)	19 (9.95%)
**Operation duration** (min)	234 (195; 276)

*^†^* Missing values.

**Table 2:** Descriptive statistics of cholesterol, HDL-C, LDL-C, and triglycerides at the different time points. Means and interquartile ranges are shown, and *p*-values indicate if the lipid values differ at the four time points. Post-hoc comparisons of pairwise differences in lipid levels are illustrated with Cliff’s delta (*d*) as effect size.

Time Point	Cholesterol (mmol/L)	HDL-C (mmol/L)	LDL-C (mmol/L)	Triglycerides (mmol/L)
**Baseline**	4.1 (3.5–5.1)	1.2 (1.01.6)	2.2 (1.7–2.9)	1.3 (1.0–2.0)
**Pre-operative**	4.3 (3.7–5.0)	1.1 (0.9–1.4)	2.7 (2.1–3.4)	1.3 (1.0–1.9)
**Intra-operative**	2.8 (2.33.4)	0.7 (0.6–0.9)	1.7 (1.3–2.2)	0.5 (0.4–0.7)
**Post-operative**	2.6 (2.2–3.3)	0.9 (0.8–1.1)	1.5 (1.1–2.0)	1.0 (0.7–1.3)
**P** *^‡^*	<0.0001	<0.0001	<0.0001	<0.0001
Post-hoc comparisons (*d* *)				
Baseline vs. Pre-operative	−0.08 (−0.20, 0.03) *^†^*	0.24 (0.12, 0.35) *^†^*	−0.33 (−0.44, −0.22) *^†^*	0.01 (−0.10, 0.13) *^†^*
Baseline vs. Intra-operative	0.73 (0.65, 0.79) *^†^*	0.82 (0.75, 0.87) *^†^*	0.33 (0.22, 0.44) *^†^*	0.86 (0.80, 0.90)
Baseline vs. Post-operative	0.74 (0.66, 0.80) *^†^*	0.63 (0.53, 0.70) *^†^*	0.45 (0.35, 0.55) *^†^*	0.41 (0.30, 0.50) *^†^*
Intra-operative vs. Post-operative	0.08 (−0.04, 0.19)	−0.39 (−0.49, −0.28) *^†^*	0.17 (0.05, 0.28) *^†^*	−0.66 (−0.74, −0.58) *^†^*
Pre-operative vs. Intra-operative	0.75 (0.68, 0.82) *^†^*	0.72 (0.64, 0.79) *^†^*	0.66 (0.57, 0.73) *^†^*	0.86 (0.81, 0.90) *^†^*
Pre-operative vs. Post-operative	0.77 (0.69, 0.82) *^†^*	0.46 (0.36, 0.55) *^†^*	0.73 (0.65, 0.79) *^†^*	0.40 (0.29, 0.50) *^†^*

*^‡^* Friedman Test; * Mean and 95% confidence intervals are shown (note that *d* is positive when the lipid levels of the first time point of the comparison are greater than the levels of the second time point and vice versa; *^†^* Statistically significant (all *p* < 0.0001) after Wilcoxon signed-rank test with a Bonferroni adjustment for multiple comparisons.

**Table 3:** Clinical outcomes. Nominal values present total number of patients for a particular clinical endpoint, with distribution of the interquartile range as a percentage.

Clinical Outcomes	All Patients
**30-day mortality**	
Died	2 (1.04%; 0.13%–3.71%)
Survived	190 (98.96%; 96.29%–99.87%)
**1-year follow-up**	
Alive	179 (96.24%; 92.40%–98.47%)
Deceased	7 (3.76%; 1.53%–7.60%)
**Hospital length of stay postoperative** (days)	7.00 (6.00; 9.00)
**Postoperative stroke**	
No	179 (94.21%; 89.88%–97.07%)
Yes	11 (5.79%; 2.93%–10.12%)
**Myocardial infarction**	
No	186 (96.88%: 93.32%–98.84%)
Yes	6 (3.12%; 1.16%–6.68%)

**Table 4:** Association between individual lipids (in units (mmol/L)) and the outcome postoperative stroke. Preoperative values as well as their change during the operation (postoperative minus preoperative) are presented. The left part of the table shows odds ratios (OR) and their 95% confidence intervals for univariable regressions, both for the crude association and adjusted for the confounders age, sex, BMI, and statins. The right part shows the OR computed by an elastic net logistic regression to account for the correlation across the lipids. An OR > 1 refers to increased risk, and an OR < 1 refers to a protective effect with respect to a unit change in a particular characteristic.

	Logistic Regression (Univariable)				Elastic Net (Multivariable)	
Covariate	OR	*P*	OR (Adjusted)	*P*	OR	OR (Adjusted)
**Cholesterol** (preop)	2.12 (1.23−3.82)	0.008	1.48 (0.74−3.17)	0.284	1.25	1.13
**Cholesterol** (∆)	0.46 (0.20−1.05)	0.062	0.29 (0.07−0.90)	0.047	0.97	0.78
**HDL-C** (preops)	3.12 (0.45−20.64)	0.239	1.95 (0.15−28.40)	0.612		0.46
**HDL-C** (∆)	0.08 (0.00−1.78)	0.104	0.01 (0.00−0.78)	0.039	0.30	0.08
**LDL-C** (preop)	2.51 (1.27−5.21)	0.010	1.68 (0.69−4.53)	0.272	1.39	1.33
**LDL-C-**(∆)	0.27 (0.08−0.91)	0.035	0.19 (0.03−0.88)	0.045	0.86	0.67
**Triglycerides** (preop)	1.03 (0.44−1.89)	0.937	0.82 (0.28−2.07)	0.699		0.91
**Triglycerides** (∆)	1.2 (0.41−5.28)	0.777	0.91 (0.25−4.14)	0.890		0.92
**Age** (y)	1.07 (1.00−1.16)	0.102				1.04
**Sex** (male)	1.13 (1.01−1.25)	0.029				0.30
**BMI** (kg/m^2^)	0.1 (0.02−0.38)	0.001				1.16
**Statin** (Yes)	0.08 (0.00−0.41)	0.015				0.22

**Table 5:** Association between individual lipids (in units (mmol/L)) and the outcome survival after one year follow-up. Preoperative values as well as their change during the operation (postoperative minus preoperative) are presented. The left part of the table shows odds ratios (OR) and their 95% confidence intervals for the case of univariable regressions both for the crude association and adjusted for the confounders age, sex, BMI, and statins. The right part shows the OR computed by an elastic net logistic regression to account for the correlation across the lipids. An OR > 1 refers to increased risk, and OR < 1 refers to a protective effect with respect to a unit change in a particular characteristic.

	Logistic Regression (Univariable)				Elastic Net	
Covariate	OR	*P*	OR (Adjusted)	*P*	OR	OR (Adjusted)
**Cholesterol** (preop.)	0.51 (0.25, 1.01)	0.058	0.45 (0.20, 0.98)	0.046	0.35	0.68
**Cholesterol** (∆)	1.81 (0.67, 4.64)	0.220	1.8 (0.67, 4.61)	0.217	4.57	1.49
**HDL-C** (preop.)	1.17 (0.09, 12.14)	0.900	0.8 (0.05, 10.46)	0.869	3.00	
**HDL-C** (∆)	0.11 (0.00, 4.79)	0.233	0.15 (0.00, 8.22)	0.334	0.01	0.16
**LDL-C** (preop.)	0.55 (0.19, 1.39)	0.239	0.5 (0.15, 1.48)	0.230		0.96
**LDL-C** (∆)	0.99 (0.22, 5.14)	0.992	0.96 (0.19, 5.21)	0.965	0.07	0.67
**Triglycerides** (preop.)	0.97 (0.29, 2.26)	0.952	1.19 (0.33, 3.08)	0.750	3.59	1.18
**Triglycerides** (∆)	1.13 (0.27, 7.66)	0.888	0.94 (0.19, 6.88)	0.948	2.06	
**Age** (y)	1.02 (0.95, 1.12)	0.585				
**Sex** (Male)	0.95 (0.79, 1.11)	0.563				0.91
**BMI** (kg/m^2^)	0.79 (0.16, 5.66)	0.783				0.98
**Statin** (Yes)	1.13 (0.24, 5.86)	0.878				0.83

## Data Availability

“MDPI Research Data Policies” at https://www.mdpi.com/ethics.

## Supplementary Materials

The following are available online at https://www.mdpi.com/article/10.3390/cells10102717/s1, Table S1: Baseline patient demographics and perioperative characteristics grouped by statin therapy; Table S2: Demographics and comorbidities stratified according to the outcomes considered in this study; Table S3: Effect of cardiopulmonary bypass time as a potential confounder for postoperative stroke; Table S4: Missing Data; Figure S5: Time series of cholesterol (a), HDL-C (b), LDL-C (c), and triglycerides (d) for the baseline, pre-operative, intra-operative and post-operative time points for those patients only who did not receive any blood transfusion perioperatively; Table S6: Descriptive statistics of cholesterol, HDL-C, LDL-C, and triglycerides at the different time points for those patients only who did not receive any blood transfusion perioperatively; Table S7: Association between individual lipids (in units [mmol/L]) and the outcome postoperative stroke.
