# Supplementary material for: Effect of Perioperative Lipid Status on Clinical Outcomes after Cardiac Surgery
Source: Cells. 2021 Oct 11;10(10):2717. doi: 10.3390/cells10102717 (PMC8534806; doi:10.3390/cells10102717)
Supplement: Supplementary file 1 [file cells-10-02717-s001.zip › cells-1368789-supplementary.pdf]

Supplementary Material

Supplementary Table S1: Baseline patient demographics and perioperative characteristics grouped by statin therapy

	All patients (N=192)	Statin: No (N=87)	Statin: Yes (N=105)	*P*
Demographics				
**Age (yr)**	67.0 [60.0;73.0]	65.0 [57.5;73.5]	68.0 [62.0;73.0]	0.128
**Height (cm)**	173 (8.71)	173 (9.79)	173 (7.74)	0.554
**Weight (kg)**	80.4 [70.0;90.1]	80.1 [68.2;88.5]	81.4 [71.0;94.5]	0.283
**BMI (kg/m²)**	26.1 [23.7;30.4]	25.7 [23.6;29.5]	26.5 [23.8;30.7]	0.291
**Sex (Male)**	145 (75.5%)	56 (64.4%)	89 (84.8%)	0.002
Comorbidities				
**Diabetes (Yes)**	35 (18.2%)	7 (8.05%)	28 (26.7%)	0.002
**Diabetes on insulin**				0.374
**No**	24 (68.6%)	6 (85.7%)	18 (64.3%)	
**Yes**	11 (31.4%)	1 (14.3%)	10 (35.7%)	
**Hypertension† (Yes)**	130 (68.4%)	47 (54.7%)	83 (79.8%)	<0.001
**Dyslipidaemia† (Yes)**	111 (58.1%)	28 (32.2%)	83 (79.8%)	<0.001
**Nicotine†**				0.060
**Former smoker**	49 (26.1%)	16 (19.0%)	33 (31.7%)	
**Non-smoker**	97 (51.6%)	51 (60.7%)	46 (44.2%)	
**Smoker**	42 (22.3%)	17 (20.2%)	25 (24.0%)	
**Obesity (Yes)**	52 (27.1%)	20 (23.0%)	32 (30.5%)	0.318
**Preoperative renal disease (Yes)**	43 (22.4%)	16 (18.4%)	27 (25.7%)	0.299
**Peripheral vascular disease†**				0.366
**No**	167 (93.8%)	75 (94.9%)	92 (92.9%)	
**Stage 1**	4 (2.25%)	1 (1.27%)	3 (3.03%)	
**Stage 2**	4 (2.25%)	1 (1.27%)	3 (3.03%)	
**Stage 3**	1 (0.56%)	0 (0.00%)	1 (1.01%)	
**Stage 4**	2 (1.12%)	2 (2.53%)	0 (0.00%)	
**Carotid disease†**				0.168
**< 50%**	1 (0.58%)	0 (0.00%)	1 (0.99%)	
**>90%**	3 (1.75%)	1 (1.43%)	2 (1.98%)	
**50-69%,**	9 (5.26%)	1 (1.43%)	8 (7.92%)	
**70-89%**	2 (1.17%)	0 (0.00%)	2 (1.98%)	
**no**	156 (91.2%)	68 (97.1%)	88 (87.1%)	
**Myocardial infarction†**				0.004
**No MI**	171 (89.5%)	85 (97.7%)	86 (82.7%)	
**MI 0-7 days before operation**	3 (1.57%)	1 (1.15%)	2 (1.92%)	
**MI 8-90 days before operation**	8 (4.19%)	0 (0.00%)	8 (7.69%)	
**MI >90 days before operation**	9 (4.71%)	1 (1.15%)	8 (7.69%)	
**COPD† (Yes)**	23 (12.1%)	10 (11.5%)	13 (12.6%)	0.989
**NYHA†**				0.254
**1**	60 (31.4%)	21 (24.4%)	39 (37.1%)	
**2**	90 (47.1%)	44 (51.2%)	46 (43.8%)	
**3**	38 (19.9%)	20 (23.3%)	18 (17.1%)	
**4**	3 (1.57%)	1 (1.16%)	2 (1.90%)	
**CCS†**				0.001
**0**	118 (62.4%)	65 (76.5%)	53 (51.0%)	
**1**	34 (18.0%)	13 (15.3%)	21 (20.2%)	
**2**	25 (13.2%)	5 (5.88%)	20 (19.2%)	
**3**	9 (4.76%)	2 (2.35%)	7 (6.73%)	
**4**	3 (1.59%)	0 (0.00%)	3 (2.88%)	
**Ejection fraction†**	60.0 [55.0;65.0]	60.0 [55.0;65.0]	60.0 [57.0;65.0]	0.565
**EuroSCORE2†**	1.73 [0.90;2.93]	2.03 [1.26;3.49]	1.44 [0.85;2.77]	0.050

Pre-operative lipids				
**Cholesterol (mmol/L)**	4.42 (1.13)	5.03 (1.04)	3.92 (0.94)	<0.001
**HDL cholesterol(mmol/L)**	1.13 [0.92;1.35]	1.20 [1.00;1.42]	1.08 [0.88;1.31]	0.012
**LDL Cholesterol (mmol/L)**	2.68 [2.14;3.40]	3.25 [2.74;3.85]	2.34 [1.98;2.70]	<0.001
**Triglycerides (mmol/L)**	1.33 [0.97;1.88]	1.26 [0.95;1.75]	1.34 [1.00;1.92]	0.323
Perioperative Characteristics				
**Aortic valve Surgery (Yes)**	86 (44.8%)	45 (51.7%)	41 (39.0%)	0.107
**Mitral valve surgery (Yes)**	45 (23.4%)	31 (35.6%)	14 (13.3%)	0.001
**Tricuspid valve surgery (Yes)**	17 (8.85%)	13 (14.9%)	4 (3.81%)	0.014
**Coronary Artery Bypass Surgery (Yes)**	77 (40.1%)	11 (12.6%)	66 (62.9%)	<0.001
**Ascending Aortic surgery (Yes)**	38 (19.8%)	28 (32.2%)	10 (9.52%)	<0.001
**Aortic Arch Surgery (Yes)**	11 (5.73%)	9 (10.3%)	2 (1.90%)	0.016
**ECC or MiECC**				<0.001
**ECC**	149 (77.6%)	85 (97.7%)	64 (61.0%)	
**MiECC**	43 (22.4%)	2 (2.30%)	41 (39.0%)	
**Bypass time (min)**	104 [80.0;132]	109 [86.0;134]	98.0 [76.0;132]	0.181
**Aortic cross clamping (min)**	68.5 [52.0;91.8]	71.0 [52.0;96.0]	67.0 [51.0;88.0]	0.527
**Lowest body temperature (deg C)**	33.2 [32.1;33.8]	32.4 [31.8;33.6]	33.5 [32.6;34.0]	<0.001
**Deep hypothermic cardiac arrest† (Yes)**	19 (9.95%)	16 (18.4%)	3 (2.88%)	0.001
**Operation duration (min)**	234 [195;276]	211 [179;248]	255 [205;286]	<0.001

Supplementary Table S2: Demographics and comorbidities stratified according to the outcomes considered in this study

	Postoperative Stroke			Myocardial Infarction			30-day survival			1-year survival		
**Characteristic**	**No**	**Yes**	***P***	**No**	**Yes**	***P***	**Alive**	**Deceased**	***P***	**Alive**	**Deceased**	***P***
	N=179	***N=11***		***N=190***	***N=2***		***N=190***	***N=2***		***N=179***	***N=7***	
**Age**	67.0 [59.5;73.0]	69.0 [62.5;77.0]	0.097	67.0 [60.0;73.0]	61.0 [60.5;61.5]	0.312	67.0 [60.0;73.0]	70.5 [69.8;71.2]	0.514	67.0 [60.0;73.0]	69.0 [62.0;70.5]	0.810
**Height**	173 (8.61)	167 (9.54)	0.066	173 (8.73)	166 (1.41)	0.028	173 (8.62)	162 (14.8)	0.496	173 (8.76)	168 (9.14)	0.222
**Weight**	80.3 [70.0;90.6]	84.3 [77.3;88.5]	0.374	80.8 [70.0;90.2]	71.5 [70.8;72.2]	0.288	80.3 [70.0;90.2]	89.0 [88.5;89.5]	0.277	81.4 [70.0;91.2]	78.5 [66.0;83.6]	0.311
**BMI**	25.9 [23.6;30.3]	28.8 [27.1;35.0]	0.054	26.1 [23.7;30.4]	25.9 [25.8;26.0]	0.939	26.0 [23.7;30.4]	34.1 [32.1;36.1]	0.091	26.1 [23.6;30.4]	25.6 [22.0;28.5]	0.545
**Sex:**			0.002			0.052			0.434			1.000
Female	39 (21.8%)	8 (72.7%)		45 (23.7%)	2 (100%)		46 (24.2%)	1 (50.0%)		43 (24.0%)	2 (28.6%)	
Male	140 (78.2%)	3 (27.3%)		145 (76.3%)	0 (0.00%)		144 (75.8%)	1 (50.0%)		136 (76.0%)	5 (71.4%)	
**Diabetes:**			0.698			1.000			0.320			0.090
No	147 (82.1%)	10 (90.9%)		155 (81.6%)	2 (100%)		156 (82.1%)	1 (50.0%)		149 (83.2%)	4 (57.1%)	
Yes	32 (17.9%)	1 (9.09%)		35 (18.4%)	0 (0.00%)		34 (17.9%)	1 (50.0%)		30 (16.8%)	3 (42.9%)	
**Hypertension:**			0.755			1.000			0.555			0.668
No	57 (32.2%)	3 (27.3%)		59 (31.4%)	1 (50.0%)		60 (31.9%)	0 (0.00%)		58 (32.6%)	1 (16.7%)	
Yes	120 (67.8%)	8 (72.7%)		129 (68.6%)	1 (50.0%)		128 (68.1%)	2 (100%)		120 (67.4%)	5 (83.3%)	
**Dyslipidaemia:**			0.205			0.512			1.000			0.700
No	72 (40.4%)	7 (63.6%)		80 (42.3%)	0 (0.00%)		79 (41.8%)	1 (50.0%)		73 (40.8%)	3 (50.0%)	
Yes	106 (59.6%)	4 (36.4%)		109 (57.7%)	2 (100%)		110 (58.2%)	1 (50.0%)		106 (59.2%)	3 (50.0%)	
**Nicotine:**			0.443			0.741			0.727			0.149
Former smoker	47 (26.9%)	1 (9.09%)		49 (26.3%)	0 (0.00%)		49 (26.3%)	0 (0.00%)		47 (26.6%)	0 (0.00%)	
Non-smoker	90 (51.4%)	7 (63.6%)		96 (51.6%)	1 (50.0%)		96 (51.6%)	1 (50.0%)		91 (51.4%)	3 (50.0%)	
Smoker	38 (21.7%)	3 (27.3%)		41 (22.0%)	1 (50.0%)		41 (22.0%)	1 (50.0%)		39 (22.0%)	3 (50.0%)	
**Obesity:**			0.159			0.607			0.086			1.000
No	133 (74.3%)	6 (54.5%)		138 (72.6%)	2 (100%)		140 (73.7%)	0 (0.00%)		130 (72.6%)	5 (71.4%)	
Yes	46 (25.7%)	5 (45.5%)		52 (27.4%)	0 (0.00%)		50 (26.3%)	2 (100%)		49 (27.4%)	2 (28.6%)	
**Preoperative renal disease:**			0.674			0.415			0.059			0.004
No	140 (78.2%)	8 (72.7%)		148 (77.9%)	1 (50.0%)		149 (78.4%)	0 (0.00%)		143 (79.9%)	2 (28.6%)	
Yes	39 (21.8%)	3 (27.3%)		42 (22.1%)	1 (50.0%)		41 (21.6%)	2 (100%)		36 (20.1%)	5 (71.4%)	
**Peripheral vascular disease:**			1.000			0.101			1.000			0.296
No	154 (93.3%)	11 (100%)		166 (94.3%)	1 (50.0%)		165 (93.8%)	2 (100%)		158 (94.0%)	4 (80.0%)	
Stadium 1	4 (2.42%)	0 (0.00%)		3 (1.70%)	1 (50.0%)		4 (2.27%)	0 (0.00%)		4 (2.38%)	0 (0.00%)	
Stadium 2	4 (2.42%)	0 (0.00%)		4 (2.27%)	0 (0.00%)		4 (2.27%)	0 (0.00%)		3 (1.79%)	1 (20.0%)	
Stadium 3	1 (0.61%)	0 (0.00%)		1 (0.57%)	0 (0.00%)		1 (0.57%)	0 (0.00%)		1 (0.60%)	0 (0.00%)	
Stadium 4	2 (1.21%)	0 (0.00%)		2 (1.14%)	0 (0.00%)		2 (1.14%)	0 (0.00%)		2 (1.19%)	0 (0.00%)	
**Carotid disease:**			0.367			0.039			1.000			0.487
< 50%	1 (0.63%)	0 (0.00%)		1 (0.59%)	0 (0.00%)		1 (0.59%)	0 (0.00%)		1 (0.63%)	0 (0.00%)	
>90%	2 (1.26%)	1 (10.0%)		3 (1.78%)	0 (0.00%)		3 (1.78%)	0 (0.00%)		3 (1.89%)	0 (0.00%)	
50-69%,	9 (5.66%)	0 (0.00%)		9 (5.33%)	0 (0.00%)		9 (5.33%)	0 (0.00%)		7 (4.40%)	1 (14.3%)	
70-89%	2 (1.26%)	0 (0.00%)		1 (0.59%)	1 (50.0%)		2 (1.18%)	0 (0.00%)		2 (1.26%)	0 (0.00%)	
no	145 (91.2%)	9 (90.0%)		155 (91.7%)	1 (50.0%)		154 (91.1%)	2 (100%)		146 (91.8%)	6 (85.7%)	
**COPD:**			1.000			0.237			0.221			0.213
No	156 (88.1%)	10 (90.9%)		166 (88.3%)	1 (50.0%)		166 (88.3%)	1 (50.0%)		156 (88.1%)	5 (71.4%)	
Yes	21 (11.9%)	1 (9.09%)		22 (11.7%)	1 (50.0%)		22 (11.7%)	1 (50.0%)		21 (11.9%)	2 (28.6%)	
**NYHA:**			0.923			1.000			0.077			0.154
1	57 (32.0%)	3 (27.3%)		59 (31.2%)	1 (50.0%)		60 (31.7%)	0 (0.00%)		56 (31.5%)	2 (28.6%)	
2	84 (47.2%)	6 (54.5%)		89 (47.1%)	1 (50.0%)		90 (47.6%)	0 (0.00%)		86 (48.3%)	1 (14.3%)	
3	34 (19.1%)	2 (18.2%)		38 (20.1%)	0 (0.00%)		36 (19.0%)	2 (100%)		33 (18.5%)	4 (57.1%)	
4	3 (1.69%)	0 (0.00%)		3 (1.59%)	0 (0.00%)		3 (1.59%)	0 (0.00%)		3 (1.69%)	0 (0.00%)	
**CCS:**			0.557			1.000			0.377			1.000
0	110 (62.1%)	8 (72.7%)		116 (62.0%)	2 (100%)		117 (62.6%)	1 (50.0%)		111 (63.1%)	5 (71.4%)	
1	31 (17.5%)	3 (27.3%)		34 (18.2%)	0 (0.00%)		34 (18.2%)	0 (0.00%)		31 (17.6%)	1 (14.3%)	
2	24 (13.6%)	0 (0.00%)		25 (13.4%)	0 (0.00%)		24 (12.8%)	1 (50.0%)		22 (12.5%)	1 (14.3%)	
3	9 (5.08%)	0 (0.00%)		9 (4.81%)	0 (0.00%)		9 (4.81%)	0 (0.00%)		9 (5.11%)	0 (0.00%)	
4	3 (1.69%)	0 (0.00%)		3 (1.60%)	0 (0.00%)		3 (1.60%)	0 (0.00%)		3 (1.70%)	0 (0.00%)	
**Ejection Fraction**	60.0 [55.0;65.0]	60.0 [54.5;62.5]	0.625	60.0 [55.0;65.0]	57.5 [53.8;61.2]	0.910	60.0 [55.0;65.0]	30.0 [30.0;30.0]	0.020	60.0 [55.0;65.0]	40.0 [30.0;62.5]	0.078

	Postoperative Stroke			Myocardial Infarction			30-day survival			1-year survival		
**Characteristic**	**No** ***N=179***	**Yes** ***N=11***	***P***	**No** ***N=190***	**Yes** ***N=2***	***P***	**Alive** ***N=190***	**Deceased** ***N=2***	***P***	**Alive** ***N=179***	**Deceased** ***N=7***	***P***
**EuroSCORE2**	1.65 [0.88;2.80]	3.69 [2.36;7.58]	0.005	1.69 [0.90;2.92]	5.87 [4.09;7.65]	0.178	1.69 [0.90;2.91]	14.1 [12.4;15.8]	0.019	1.76 [0.90;2.91]	1.96 [1.35;7.85]	0.281
**Logistic EuroSCORE**	4.50 [2.29;7.44]	17.4 [6.56;22.1]	0.001	4.65 [2.33;7.94]	8.46 [7.77;9.15]	0.222	4.65 [2.33;7.94]	11.1 [8.52;13.7]	0.207	4.65 [2.36;8.19]	5.92 [3.37;11.3]	0.541
**Additive EuroSCORE**	5.00 [3.00;7.00]	10.0 [6.50;11.0]	0.001	6.00 [3.00;7.00]	7.50 [7.25;7.75]	0.217	6.00 [3.00;7.00]	7.50 [6.75;8.25]	0.278	6.00 [3.00;7.50]	6.00 [4.00;8.00]	0.702
**Statin:**			0.005			1.000			1.000			1.000
No	77 (43.0%)	10 (90.9%)		86 (45.3%)	1 (50.0%)		86 (45.3%)	1 (50.0%)		82 (45.8%)	3 (42.9%)	
Yes	102 (57.0%)	1 (9.09%)		104 (54.7%)	1 (50.0%)		104 (54.7%)	1 (50.0%)		97 (54.2%)	4 (57.1%)	

Supplementary Table S3: Effect of cardiopulmanory bypass time as a potential confounder for postoperative stroke

	**Logistic Regression (univariable)**				**Elastic net (multivariable)**		**Elastic net (multivariabe; with additional bypass time as confounder)**	
**Covariate**	**OR**	***P***	**OR (adjusted)**	***P***	**OR**	**OR (adjusted)**	**OR**	**OR (adjusted)**
**Cholesterol** (preops)	2.12 (1.23, 3.82)	0.008	1.48 (0.74, 3.17)	0.284	1.25	1.13	1.25	1.24
**Cholesterol** (Δ)	0.46 (0.20, 1.05)	0.062	0.29 (0.07, 0.90)	0.047	0.97	0.78	0.97	0.75
**HDL Cholesterin** (preops)	3.12 (0.45, 20.64)	0.239	1.95 (0.15, 28.40)	0.612	-	0.46		0.30
**HDL Cholesterin** (Δ)	0.08 (0.00, 1.78)	0.104	0.01 (0.00, 0.78)	0.039	0.30	0.08	0.30	0.08
**LDL Cholesterin** (preops)	2.51 (1.27, 5.21)	0.010	1.68 (0.69, 4.53)	0.272	1.39	1.33	1.39	1.44
**LDL Cholesterin** (Δ)	0.27 (0.08, 0.91)	0.035	0.19 (0.03, 0.88)	0.045	0.86	0.67	0.86	0.95
**Triglyceride** (preops)	1.03 (0.44, 1.89)	0.937	0.82 (0.28, 2.07)	0.699	-	0.91		0.67
**Triglyceride** (Δ)	1.2 (0.41, 5.28)	0.777	0.91 (0.25, 4.14)	0.890	-	0.92		0.68

Supplementary Table S4: Missing Data

Variable	*Number of missing values (% of total)*
**Age** (yr)	0 (0.00%)
**Height** (cm)	0 (0.00%)
**Weight** (kg)	0 (0.00%)
**BMI** (kg/m^2)	0 (0.00%)
**Sex**	0 (0.00%)
**Diabetes**	0 (0.00%)
**Diabetes on insulin**	157 (81.8%)
**Hypertension**	2 (1.04%)
**Dyslipidaemia**	1 (0.52%)
**Nicotine**	4 (2.08%)
**Adipositas**	0 (0.00%)
**Preoperative renal disease**	0 (0.00%)
**Peripheral vascular disease**	14 (7.29%)
**Carotid disease**	21 (10.9%)
**Myocardial infarction**	1 (0.52%)
**COPD**	2 (1.04%)
**NYHA**	1 (0.52%)
**CCS**	3 (1.56%)
**Ejection fraction**	1 (0.52%)
**EuroSCORE2**	8 (4.17%)
**Logistic EuroSCORE**	5 (2.60%)
**Aortic valve**	0 (0.00%)
**Mitral valve**	0 (0.00%)
**Tricuspid valve**	0 (0.00%)
**Coronary Artery Bypass**	0 (0.00%)
**Ascending Aortic**	0 (0.00%)
**Aortic Arch**	0 (0.00%)
**Cholesterol** (mmol/L)	0 (0.00%)
**HDL cholesterol**(mmol/L)	0 (0.00%)
**LDL Cholesterol** (mmol/L)	0 (0.00%)
**Quotient overall** (.)	0 (0.00%)
**Triglycerides** (mmol/L)	0 (0.00%)
**Lipoprotein(a)** (mg/dl)	0 (0.00%)
**ECC or MiECC**	0 (0.00%)
**Bypass time** (min)	0 (0.00%)
**Aortic cross clamping** (min)	0 (0.00%)
**Lowest body temperature** (deg C)	0 (0.00%)
**Deep hypothermic cardiac arrest**	1 (0.52%)
**Operation duration** (min)	0 (0.00%)

Supplementary Figure S5: Time series of cholesterol (a), HDL-C (b), LDL-C (c), and trigylcerides (d) for the baseline, pre-operative, intra-operative and post-operative time points *for those patients only who did not receive any blood transfusion perioperatively*. Shown are medians and interquartile ranges at the different time points (black dots and black lines) and time series of individual patients (grey lines).

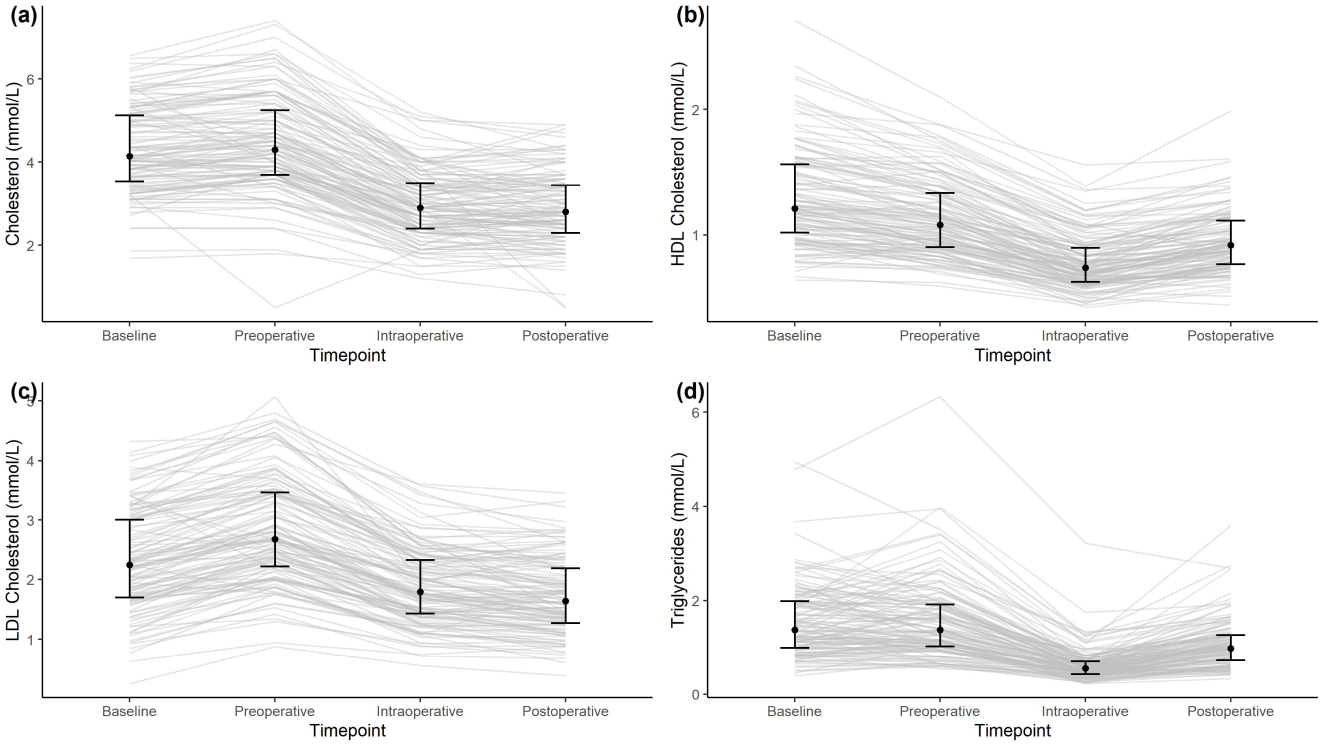

Supplementary Table S6: Descriptive statistics of cholesterol, HDL-C, LDL-C, and triglycerides at the different time points *for those patients only who did not receive any blood transfusion perioperatively*. Means and interquartile ranges are shown and P values indicate if the lipid values differ at the four time points. Post-hoc comparisons of pairwise differences in lipid levels are illustrated with Cliff's delta (d) as effect size.

Time point	Cholesterol (mmol/L)	HDL-C (mmol/L)	LDL-C (mmol/L)	Triglycerides (mmol/L)
**Baseline**	4.1 (3.5, 5.1)	1.2 (1.0, 1.6)	2.2 (1.7, 3.0)	1.4 (1.0, 2.0)
**Pre-operative**	4.3 (3.7, 5.2)	1.1 (0.9, 1.3)	2.7 (2.2, 3.5)	1.4 (1.0, 1.9)
**Intra-operative**	2.9 (2.4, 3.5)	0.7 (0.6, 0.9)	1.8 (1.4, 2.3)	0.6 (0.4, 0.7)
**Post-operative**	2.8 (2.3, 3.5)	0.9 (0.8, 1.1)	1.6 (1.3, 2.2)	1.0 (0.7, 1.3)
**P‡**	<0.0001	<0.0001	<0.0001	<0.0001
**Post-hoc comparisons (*d**)**				
**Baseline vs. Pre-operative**	-0.08 (-0.21, 0.06) †	0.25 (0.11, 0.38) †	-0.32 (-0.45, -0.19) †	0.00 (-0.14, 0.14) †
**Baseline vs. Intra-operative**	0.69 (0.58, 0.77) †	0.80 (0.71, 0.86) †	0.29 (0.15, 0.42) †	0.84 (0.75, 0.89)
**Baseline vs. Post-operative**	0.70 (0.60, 0.78) †	0.58 (0.46, 0.68) †	0.41 (0.27, 0.53) †	0.41 (0.27, 0.53) †
**Intra-operative vs. Post-operative**	0.05 (-0.09, 0.19)	-0.42 (-0.53, -0.28) †	0.15 (0.01, 0.29) †	-0.63 (-0.72, -0.51) †
**Pre-operative vs. Intra-operative**	0.72 (0.61, 0.80) †	0.68 (0.57, 0.76) †	0.61 (0.50, 0.71) †	0.85 (0.78, 0.91) †
**Pre-operative vs. Post-operative**	0.73 (0.63, 0.81) †	0.37 (0.24, 0.49) †	0.69 (0.59, 0.77) †	0.42 (0.29, 0.54) †

‡ Friedman Test; * Mean and 95% confidence intervals are shown (note that *d* is positive when the lipid levels of the first time point of the comparison are greater than the levels of the second time point, and *vice versa;* † Statistically significant (all p<0.0001) after Wilcoxon signed-rank test with a Bonferroni adjustment for multiple comparisons.

Supplementary Table S7: Association between individual lipids (in units [mmol/L]) and the outcome *postoperative stroke*. Preoperative values as well as their change during the operation (postoperative minus preoperative) are presented. The results from a multivariable (crude an adjusted to age, sex, BMI and statin) elastic net regression are presented for the entire cohort and *for those patients only who did not receive any blood transfusion perioperatively.* An OR>1 refers to increased risk and an OR<1 refers to a protective effect with respect to a unit change in a particular characteristic.

	**Elastic Net (entire cohort: N=192)**		**Elastic Net (no blood transfusion N=127)**	
**Covariate**	**OR**	**OR (adjusted)**	**OR**	**OR (adjusted)**
**TC** (preop)	1.25	1.13	1.37	1.33
**TC** (Δ)	0.97	0.78	-	0.91
**HDL-C** (preops)	-	0.46	1.14	1.30
**HDL-C** (Δ)	0.30	0.08	-	-
**LDL-C** (preop)	1.39	1.33	1.54	1.47
**LDL-C-**(Δ)	0.86	0.67	0.77	0.70
**TG** (preop)	-	0.91	-	0.99
**TG** (Δ)	-	0.92	1.12	1.09
**Age** (yr)	-	1.04	-	-
**Sex** (male)	-	0.30	-	0.67
**BMI** (kg/m^2)	-	1.16	-	1.13
**Statin** (Yes)	-	0.22	-	0.54
